# The Role of Plain Radiography in Diagnosing Brodie's Abscess: Clinical Image

**DOI:** 10.1002/ccr3.70045

**Published:** 2024-12-31

**Authors:** Ujjwal Prakash Khanal, Mitu Sadashankar, Siddhartha Bhandari

**Affiliations:** ^1^ Tribhuwan University Institute of Medicine Kathmandu Nepal; ^2^ Nobel Medical College and Teaching Hospital Kathmandu University Nepal; ^3^ Department of Radiology Newark Beth Israel Medical Center New Jersey USA

**Keywords:** bone infections, Brodie's abscess, plain radiography, subacute osteomyelitis

## Abstract

Plain radiographs can still be of considerable diagnostic value for recognizing Brodie's abscess, especially in resource constrained settings.

## 1 Case Presentation

**FIGURE 1:**
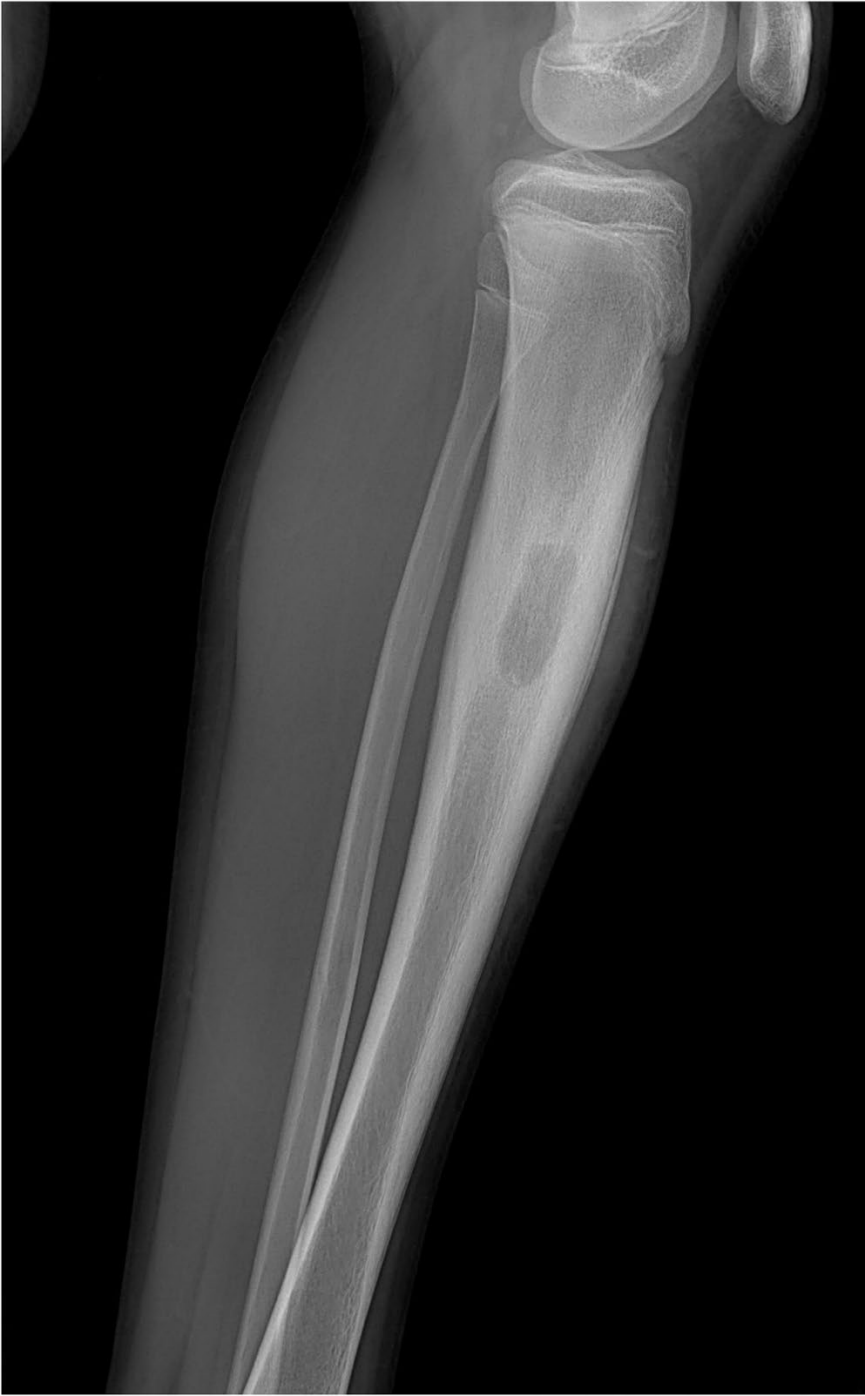
Plain radiograph showing lateral view of the leg. The radiolucent abscess cavity can be seen in the diaphysis with adjacent sclerosis and periosteal reaction. *[Dr. Ujjwal Prakash Khanal, Primary Care Physician]*.

**FIGURE 2:**
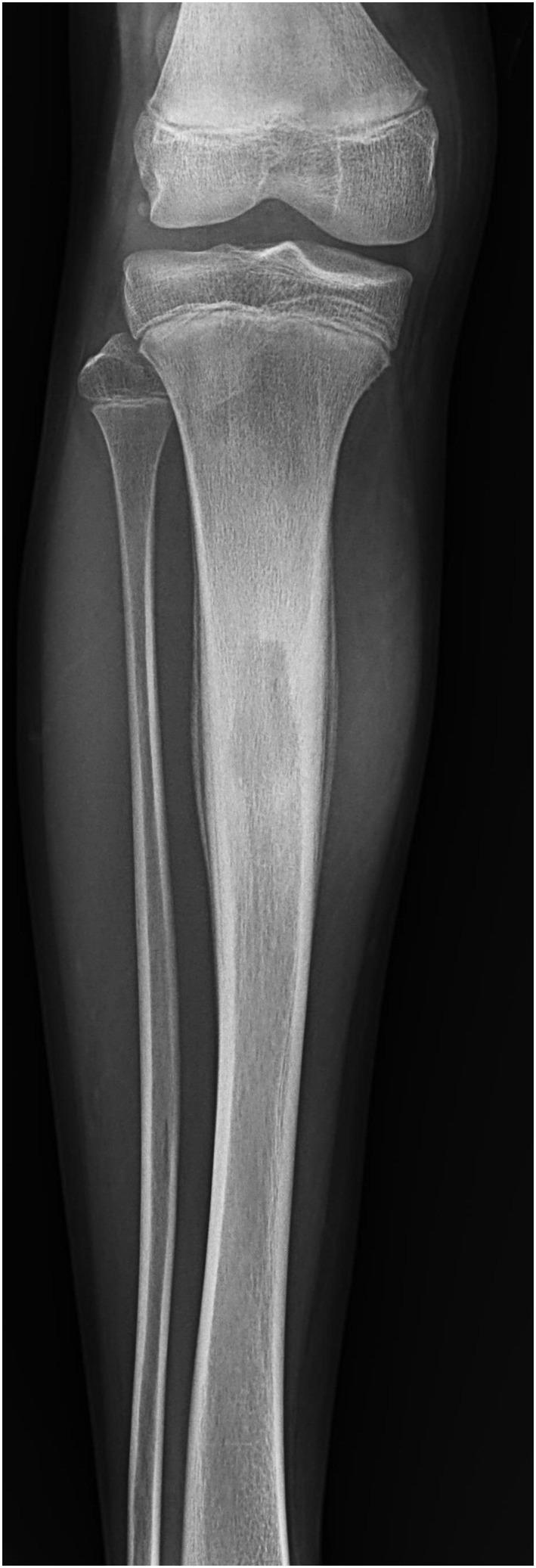
Plain radiograph showing anteroposterior view of the leg. The abscess cavity is surrounded by reactive sclerosis and periosteal reaction on both sides. *[Dr. Ujjwal Prakash Khanal, Primary Care Physician]*.

**FIGURE 3:**
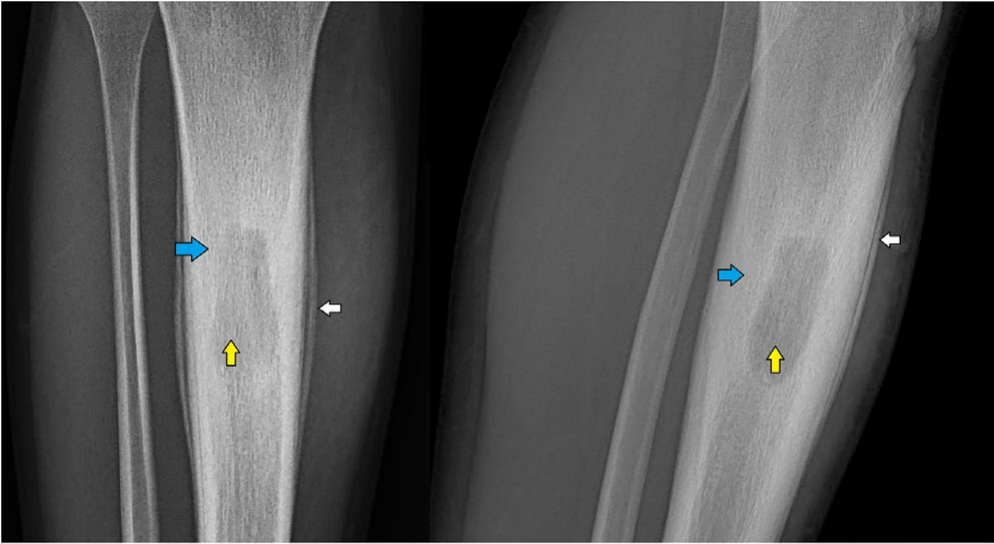
Zoomed in versions of the radiographs in Figure 1 and Figure 2. The arrows highlight periosteal reaction (white), the abscess cavity (yellow) and the reactive sclerosis (blue). *[Dr. Ujjwal Prakash Khanal, Primary Care Physician]*.

A 12‐year‐old girl presented to the outpatient department with insidious onset of pain in the right leg for four weeks. She had presented to a local clinic with the complaint earlier, but had been returned with over‐the‐counter NSAIDs without further diagnostic workup. Physical examination did not reveal any abnormality except for warmth over the proximal portion of the right leg. Complete blood count and serum markers of inflammation were all normal. The plain radiographs, however, showed a clear elongated radiolucency in the diaphysis surrounded by dense reactive sclerosis (Figures 1, 2, 3). Distinct periosteal reaction could also be identified in the images. A diagnosis of Brodie's abscess was thus made and intravenous antibiotics were started while planning for surgical drainage.

## 2 Discussion

Brodie's abscess is a subacute form of osteomyelitis characterized by intraosseous collection of pus, usually involving the tibia and the femur [1]. Diagnosing this uncommon condition can be quite difficult since clinical features are vague and laboratory markers of inflammation, like the total leucocyte count, erythrocyte sedimentation rate and C‐Reactive Protein, are frequently normal [2]. As such, higher imaging modalities, like MRI or CT, are often emphasized for diagnosis [1]. However, when ordered and interpreted with a reasonable index of suspicion, plain radiography findings can also be distinctive and typically include a well demarcated, elongated and medullary‐based lytic lesion with a sclerotic rim, sometimes associated with a periosteal reaction indicative of regenerative processes that occur concurrently with subacute and chronic osteomyelitis [2, 3].

There are other conditions that may present with lytic bone lesions, but they can generally be distinguished from Brodie's abscess with associated findings, like an onion‐skin periosteal reaction in Ewing sarcoma, epiphyseal location with multicystic appearance in Aneurysmal bone cysts and giant cell tumors, extensive cortical destruction or a moth‐eaten appearance in malignancies, an eccentric, cortical location in osteoid osteoma, and multifocal lytic lesions in chronic recurrent multifocal osteomyelitis (CRMO) [2, 3]. As such, while radiographic findings do not negate the need for further pathological workup, our case demonstrates that plain radiographs alone may sometimes suffice as the imaging modality of choice to robustly establish a provisional diagnosis of Brodie's abscess, potentially reducing diagnostic time and costs [1]. This could be significant for many hospitals that do not have access to more sophisticated imaging modalities, particularly in regions where the incidence of osteomyelitis is high.

## Author Contributions

**Ujjwal Prakash Khanal:** conceptualization, investigation, writing (original draft). **Mitu Sadashankar:** writing (original draft). **Siddhartha Bhandari:** writing (review and editing).

## Ethics Statement

This study is in compliance with the declaration of Helsinki.

## Consent

Written informed consent was obtained from the patient for publication of this case report in accordance with the journal's patient consent policy.

## Conflicts of Interest

The authors declare no conflicts of interest.

## Data Availability

All data generated or analyzed during this study are included in this published article.
